# Factors associated with initiation and continuation of endocrine therapy in women with hormone receptor-positive breast cancer

**DOI:** 10.1186/s12885-022-09946-x

**Published:** 2022-08-01

**Authors:** Beomyoung Cho, Maria Pérez, Donna B. Jeffe, Matthew W. Kreuter, Julie A. Margenthaler, Graham A. Colditz, Ying Liu

**Affiliations:** 1 Department of Public Health, University of North Florida, Jacksonville, FL USA; 2 Department of Medicine, Washington University School of Medicine, St. Louis, MO USA; 3 Alvin J. Siteman Cancer Center at Barnes-Jewish Hospital and Washington University School of Medicine, St. Louis, MO USA; 4 The Brown School, Washington University, Saint Louis, MO USA; 5 Department of Surgery, Washington University School of Medicine, St. Louis, MO USA

**Keywords:** Breast cancer, Hormone therapy, Diabetes, Menopausal symptoms, Obesity, Depression

## Abstract

**Background:**

Despite benefits of endocrine therapy (ET) for patients with hormone-receptor (HR)-positive breast cancer, many patients do not initiate or discontinue ET against recommendations.

**Methods:**

We identified variables associated with ET initiation and continuation, analyzing pooled data from two longitudinal studies at a National Cancer Institute comprehensive cancer center in St. Louis, Missouri. The sample included 533 women with newly diagnosed, non-metastatic, HR-positive breast cancer who completed interviews at enrollment and 6, 12, and 24 months after definitive surgical treatment. Logistic regression models estimated the adjusted odds ratio and 95% confidence interval (aOR [95% CI]) for each of self-reported ET initiation by the 12-month interview and continuation for ≥12 months by the 24-month interview in association with self-reported diabetes, elevated depressed mood, menopausal-symptom severity and obesity, adjusting for race, age, insurance status, chemotherapy, and radiation therapy.

**Results:**

Overall, 81.4% (434/533) of patients initiated ET, and 86.5% (371/429) continued ET ≥12 months. Patients with diabetes had lower odds of initiating ET (0.50 [0.27-0.91]). Patients reporting greater menopausal-symptom severity had lower odds of continuing ET (0.72 [0.53-0.99]).

**Conclusion:**

Efforts to increase ET initiation among patients with diabetes and better manage severe menopausal symptoms among ET users might promote ET continuation.

**Clinical trial information:**

*ClinicalTrials.gov**: #NCT00929084.*

**Supplementary Information:**

The online version contains supplementary material available at 10.1186/s12885-022-09946-x.

## Background

About 83% of breast cancer patients in the U.S. have hormone receptor (HR)-positive (i.e., estrogen and/or progesterone receptor-positive) tumors and are eligible for endocrine therapy (ET) [1]. ET significantly reduces risks of breast cancer recurrence and mortality in conjunction with long-term adherence to oral medication [2, 3]. However, many patients do not initiate ET or discontinue use before completing the recommended minimum duration of 5 years [2–6]. In previous studies, 20-30% of HR-positive breast cancer patients did not initiate ET in the first year after diagnosis [2, 4, 6], and up to 51% of users discontinued ET prior to the recommended 5-year treatment duration [5, 6]. Thus, further investigation is needed to identify risk factors for non-initiation and/or discontinuation of ET.

Multiple variables have been associated with breast cancer patients’ ET initiation and/or continuation, such as sociodemographic characteristics [2, 7, 8], comorbidities [2, 7, 9–12], and psychosocial symptoms [6, 13–17]. Being non-Hispanic black [2], having lower socioeconomic status [2, 7], and having public or no insurance [8] are risk factors for ET underutilization. Among patients who received ET, those ≥65 years old were more likely to discontinue ET before completing the 5-year treatment compared to patients 50-64 years old [7].

Greater comorbidity-related burden also was related to higher risk of discontinuation of ET [2, 7, 12] due to comorbidity-related complications [10, 11]. Diabetes is a common comorbidity in breast cancer patients and has been associated with a lower likelihood of receiving chemotherapy and radiation therapy [9]. Obesity is another common comorbidity among patients with breast cancer [18, 19] and reduces the effectiveness of ET [20–22]. Previous studies reported an interaction between obesity and tamoxifen, increasing the risk of developing endometrial cancer [23, 24]. In addition, bipolar depression was inversely associated with ET initiation [6]. Several studies documented that patients with more severe depressive symptoms were less likely to continue ET compared with patients having less severe or no depressive symptoms [13, 14, 17]. Menopausal symptoms also have been associated with discontinuation of ET [15, 16].

However, little is known about the associations of co-existing diabetes and obesity with ET initiation and continuation. Moreover, studies examining the associations of depressed mood and menopausal symptoms with ET were limited by including only older patients (age ≥ 68 years) [6], participants in a randomized controlled trial (RCT) of aromatase inhibitors [14], a lack of racial/ethnic diversity with predominantly white (89-94%) [6, 14, 17], or small (*n* = 196) samples [16]. To fill these knowledge gaps, we examined associations of diabetes, obesity, depressed mood, and menopausal symptoms with initiation and continuation of ET using pooled data from two prospective studies (described below) to acquire a larger, more sociodemographically and socioeconomically diverse sample of women who had been diagnosed with HR-positive breast cancer. We hypothesized that having diabetes, being obese, and reporting elevated depressed mood and greater menopausal-symptom severity are inversely associated with initiation and continuation of ET, adjusting for demographic and clinical variables.

## Methods

### Study sample

**Fig. 1:**
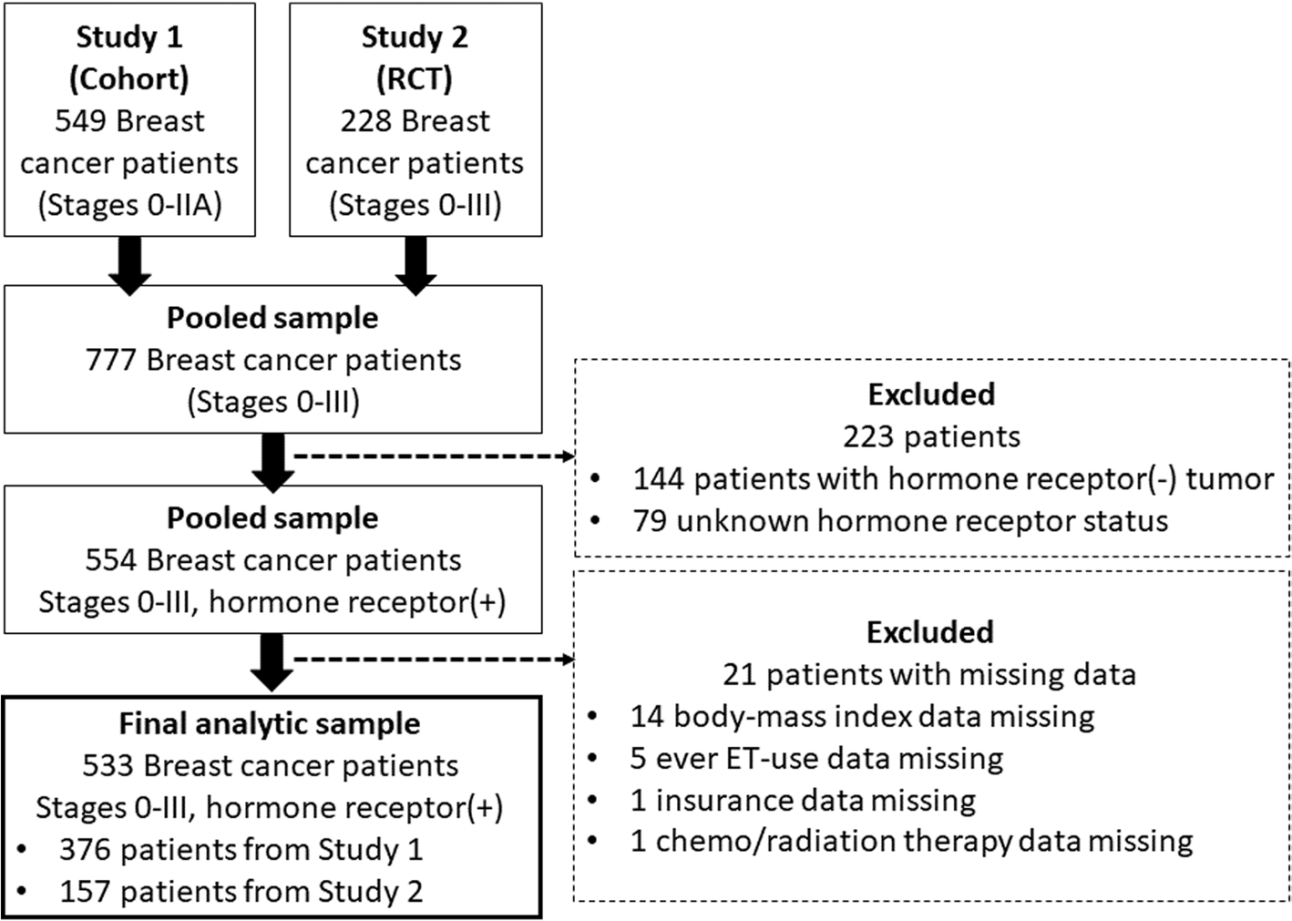
Inclusion/exclusion procedure of study sample

We conducted a secondary analysis of pooled, longitudinal data from two studies (a cohort study and an RCT) examining quality-of-life outcomes among prospectively recruited English-speaking women with newly diagnosed, first-primary, non-metastatic breast cancer, who were receiving treatment at the Siteman Cancer Center at Barnes-Jewish Hospital and Washington University School of Medicine and at Saint Louis University School of Medicine, both in St. Louis, Missouri, USA. The cohort study included 549 patients with early-stage breast cancer (Stages 0–IIA), who enrolled in the study between October 2003 and June 2007 [25]. The second study included 228 black women with non-metastatic breast cancer (Stages 0–III), who enrolled between December 2009 and 2012 in an RCT testing an interactive, cancer-communication intervention [26]. Both parent studies included multiple interviews over a 2-year follow-up period, with quality of life as the primary outcome [25]. Secondary outcomes of the RCT were adherence to recommended surveillance mammography and endocrine therapy. Patients ≥40 years old were eligible for the cohort study; black patients ≥30 years old were eligible for the second study. In both parent studies, potential participants ≥65 years old had been screened for cognitive impairment, and women with weighted scores > 10 on the Orientation-Memory-Concentration Test [27] were excluded. Thus, 549 patients in the cohort study and 228 patients in the RCT comprised the pool of patients from which this study sample was drawn (Fig. 1).

The RCT intervention used videos of black breast cancer survivor stories programmed on a touchscreen tablet-computer (clinicaltrials.gov Trial Number NCT00929084, 06/24/2009) [28]. Further information regarding the RCT, including the full protocol, in compliance with CONSORT guidelines, has been published [29]. Participants received either standard of care for their breast cancer or standard of care plus the video intervention, featuring 207 video clips of black breast cancer survivors telling their stories of living with breast cancer. Stories focused on treatment side effects, coping, social support, healthcare experiences, follow-up care, and quality of life, and could be selected for viewing by story topic or by storyteller [28].

This study was conducted in accordance with the ethical standards of the 1964 Helsinki declaration and its later amendments. Following Institutional Review Board approval at Washington University School of Medicine (WUSM IRB #201102380) and receipt of patients’ informed consent, participants completed a baseline interview 4-6 weeks following definitive surgical treatment (cohort study) or near patients’ surgical post-operative visit or the start of neoadjuvant treatment (RCT). Follow-up interviews in both studies were completed at 6, 12, and 24 months following definitive surgical treatment.

### Independent variables: diabetes, obesity, depressed mood, and menopausal symptoms

We used Katz’s validated interview adaptation [30] of the Charlson Comorbidity Index [31] to identify patients at each interview who reported having diabetes that was treated by any of diet modification, oral medication, or insulin. Substantial to near-perfect agreement [32] between self-reported diabetes and the medical-record data has been reported, with kappas ranging from 0.76 to 0.93 across several studies [33–37]. Body mass index (BMI) at each interview was calculated as a ratio of weight to height (kg/m^2^). We compared obese patients with BMI ≥ 30 kg/m^2^ to non-obese patients with BMI < 30 kg/m^2^ [38]. Depressed mood was measured at each interview using the 20-item Center for Epidemiologic Studies Depression (CESD) Scale [39]. Participants rated their experience of depressive symptoms during the past week from “rarely or none of the time” (0) to “most or all of the time” (3). We compared patients with depressed mood scores ≥16, which is indicative of elevated depressed mood, to patients with scores < 16 [39, 40]. Menopausal symptoms were measured using a previously validated 4-item measure [41]. Patients reported the severity hot flashes, cold sweats, night sweats, and vaginal dryness in the past month, ranging from (1) “not at all” to (5) “very much.” An average score of the four menopausal symptoms at each interview was computed. We also calculated the change in menopausal-symptom severity mean scores 12-18 months after patient’s first reported use of ET.

### Outcome variables

The timing of ET initiation and continuation were based on patients’ self-reported use at each interview. Near-perfect agreement [32] between the medical record and self-reported receipt of adjuvant ET was previously reported for patients in the parent cohort study (*kappa* = 0.94) [42]. ET initiation was determined using patients’ affirmative response to the question, “Did you ever take tamoxifen or other forms of endocrine/hormone therapy?” Patients who first reported “yes” to this question at baseline, 6-month, or 12-month interview were considered to have initiated ET. ET continuation was assessed based on patients’ affirmative response to ever using ET and to the question, “Are you still taking tamoxifen or other forms of endocrine/hormone therapy?” Patients were considered to have continued ET for at least 12 months if they 1) first reported ever use at baseline and reported current use at the 12-month interview, or 2) first reported ever use at the 6-month or 12-month interview and reported current use at the 24-month interview. Patients who reported ET use at baseline but not current use at the 12-month interview, and patients who reported ET use at either 6-month or 12-month interview but not current use at the 24-month interview were considered to have discontinued use.

### Covariates

Covariates previously shown to be associated with ET use included patients’ race (white or black/Asian/Pacific Islander/unspecified), insurance status (private or other, including public insurance, self-pay, or no insurance), age at enrollment (continuous), and receipt of chemotherapy (yes or no) and radiation therapy (yes or no) during the first year of the study [2, 7, 8]. Since all patients in the RCT and all patients with locally advanced breast cancer were black, and since race was a covariate of interest in this study, we did not include study type or stage in the analysis to avoid overfitting the data.

### Statistical analysis

Descriptive statistics are presented by whether or not patients initiated and continued ET. Group differences in continuous variables were tested using t-tests, and differences in categorical variables were tested with Chi-square tests. Logistic regression analysis was used to estimate the adjusted odd ratio (aOR) and 95% of confidence interval (CIs) of *ET initiation by the 12-month interview* for each of obesity, diabetes, depressed mood, and menopausal-symptom severity, adjusting for covariates. Measures of obesity, diabetes, elevated depressed mood, and menopausal symptoms at the interview *prior to* patients’ first reported use of ET were included in the analysis of ET initiation; for patients who reported initiating ET at baseline or did not initiate ET by the 12-month interview, these measures at baseline were entered. Among patients who initiated ET by the 12-month interview, logistic regression analysis was used to estimate the aOR of *ET continuation* for each of obesity, diabetes, depressed mood, and menopausal-symptom severity measured at the interview *when ET use was first reported,* and the change in menopausal-symptom severity scores 12-18 months after first report of ET initiation, adjusting for the same covariates. Statistical analyses were performed using SAS 9.4 (SAS Institute, Cary, NC). Statistical significance was assessed as two-sided *P <* 0.05.

## Results

Since one of the studies was an RCT, we first determined whether the intervention had an effect on ET initiation and continuation. There were no significant differences between the intervention and control arms in the distributions of the variables (Supplementary Table 1) or in the likelihood of ET initiation and of ET continuation adjusted for all covariates and independent variables of interest (Supplementary Table 2). In addition, there were no significant differences between the two studies in the proportions of black/other (cohort study) or black (RCT) patients with HR-positive tumors eligible for inclusion in the pooled sample (65/110 [59.1%] cohort study vs. 157/228 [68.9%] RCT, *P* = 0.08), or their proportions in association with each of the outcomes and other variables of interest in this study, except for insurance, radiation therapy, and elevated depressed mood (Table 1). Moreover, the odds of ET initiation and continuation among patients in the cohort study for exposure variables and covariates (Supplementary Table 3) were similar to findings for patients in the RCT (Supplementary Table 2), except that patients in the RCT who reported greater menopausal-symptom severity were more likely to initiate ET. Therefore, pooled data for all eligible, prospectively recruited participants in both studies were included to increase the sample size and diversity.

**Table 1:** Patient characteristics of racial minorities by the study samples

	Cohort Black/other	RCT Black	*P* value^d^
Total	65	157	
Age, years			0.08
Mean (SD)	58.9 (11.4)	56.2 (10.0)	
Insurance status			< 0.01
Private	42 (64.6%)	67 (42.7%)	
Public only or other^a^	23 (35.4%)	90 (57.3%)	
Chemotherapy			0.08
No	47 (72.3%)	94 (59.9%)	
Yes	18 (27.7%)	63 (40.1%)	
Radiation therapy			< 0.01
No	28 (43.1%)	35 (22.3%)	
Yes	37 (56.9%)	122 (77.7%)	
Diabetes			0.05
No	54 (83.1%)	111 (70.7%)	
Yes	11 (16.9%)	46 (29.3%)	
Obesity			0.80
No (BMI < 30 kg/m^2^)	26 (40.0%)	60 (38.2%)	
Yes (BMI ≥ 30 kg/m^2^)	39 (60.0%)	97 (61.8%)	
Elevated depressed mood			0.01
No (CESD < 16)	53 (81.5%)	101 (64.3%)	
Yes (CESD ≥16)	12 (18.5%)	56 (35.7%)	
Menopausal-symptom severity, mean (SD)	1.8 (0.9)	1.9 (0.9)	0.46
ET initiation^b^			0.32
No	11 (16.9%)	36 (22.9%)	
Yes	54 (83.1%)	121 (77.1%)	
ET continuation^c^			0.10
No	5 (9.4%)	23 (19.3%)	
Yes	48 (90.6%)	96 (80.7%)	

*RCT* randomized controlled trial, *ET* endocrine therapy, *SD* standard deviation, *BMI* body mass index, *CESD* Center for Epidemiologic Studies Depression scale

^a^ Other insurance status includes no insurance or self-pay

^b^ ET initiated by the 12-month interview

^c^ ET continued for at least 12 months after initiation. Of 175 patients of racial minorities who initiated ET within first 12 months, three were excluded due to missing data for obesity (*n* = 2, one from the cohort study and the other one from the RCT) and diabetes (*n* = 1 from the RCT)

^d^ Differences between the cohort study and the RCT in continuous variables were tested with the independent-samples t-test, and differences in categorical variables were tested with Chi-square test

The final sample selection included 533 patients with HR-positive tumors regardless of human epidermal growth factor receptor-2 status (Fig. 1). Only four patients were Hispanic (one white patient and three black patients), so we examined patients’ self-reported race regardless of Hispanic ethnicity. In addition, two patients were Asian Indian/Pakistani, two were Asian/Pacific Islander, and two did not respond to this question. There were no significant differences in the associations observed between each of the independent variables of interest and each outcome, ET initiation and continuation, in models that excluded these six patients (Supplementary Table 4) or combined them with black patients in one category (Tables 2 and 3). Thus, we report findings for the black/other race category that combined these six patients with black patients for analysis to increase statistical power.

**Table 2:** Characteristics of the pooled sample and prevalence of ET initiation by the 12-month interview and ET continuation for at least 12 months after initiation among hormone receptor-positive breast cancer patients

	ET initiation^a^				ET continuation^b^			
Independent variables	Total N	Yes n (%)	No n (%)	*P*^c^	Total n	Yes n (%)	No n (%)	*P*^c^
Total	533	434 (81.4)	99 (18.6)		410	370 (90.2)	40 (9.8)	
Race								
White	311	259 (83.3)	52 (16.7)	0.19	248	226 (91.1)	22 (8.9)	0.45
Black/Other^d^	222	175 (78.8)	47 (21.2)		162	144 (88.9)	18 (11.1)	
Age, years								
Mean (SD)	58.1 (10.6)	57.8 (10.2)	59.5 (11.8)	0.15	57.8 (10.2)	57.7 (10.3)	59.3 (9.3)	0.32
Insurance status								
Private	389	320 (82.3)	69 (17.7)	0.41	307	279 (90.9)	28 (9.1)	0.45
Public only or other^e^	144	114 (79.2)	30 (20.8)		103	91 (88.4)	12 (11.6)	
Chemotherapy								
No	380	293 (77.1)	87 (22.9)	<.01	275	246 (89.4)	29 (10.6)	0.44
Yes	153	141 (92.2)	12 (7.8)		135	124 (91.8)	11 (8.2)	
Radiation Therapy								
No	165	108 (65.4)	57 (34.6)	<.01	101	89 (88.1)	12 (11.9)	0.41
Yes	368	326 (88.6)	42 (11.4)		309	281 (90.9)	28 (9.1)	
Diabetes								
No	447	373 (83.4)	74 (16.6)	<.01	350	317 (90.6)	33 (9.4)	0.59
Yes	86	61 (70.9)	25 (29.1)		60	53 (88.3)	7 (11.7)	
Obesity								
No (BMI < 30 kg/m^2^)	307	249 (81.1)	58 (18.9)	0.83	238	215 (90.3)	23 (9.7)	0.94
Yes (BMI ≥ 30 kg/m^2^)	226	185 (81.9)	41 (18.1)		172	155 (90.1)	17 (9.9)	
Elevated depressed mood								
No (CESD < 16)	422	343 (81.3)	79 (18.7)	0.87	339	311 (91.7)	28 (8.3)	0.03
Yes (CESD ≥16)	111	91 (82.0)	20 (18.0)		71	59 (83.1)	12 (16.9)	
Menopausal-symptom severity, mean (SD)	1.8 (0.9)	1.8 (0.9)	1.6 (0.8)	0.04	2.1 (0.9)	2.0 (0.9)	2.4 (1.1)	0.02
Change in menopausal-symptom severity^f^, mean (SD)	–	–	–	–	0.05 (0.75)	0.07 (0.72)	−0.08 (0.95)	0.37

*ET* endocrine therapy, *SD* standard deviation, *BMI* body mass index, *CESD* Center for Epidemiologic Studies Depression scale

^a^ Using diabetes, obesity, elevated depressed mood, and menopausal symptoms from the interview prior to first report of ET use. For non-initiators and patients who first reported ever taking ET at baseline, the baseline measures of these four variables were used

^b^ Using diabetes, obesity, elevated depressed mood, and menopausal symptom data from the interview when ET initiation was first reported. Of 434 patients who initiated ET within first 12 months of the study, five were excluded due to missing data for obesity and diabetes, and 19 were excluded due to missing data for menopausal symptom severity at follow-up interviews

^c^ Group differences were tested using independent *t-*tests for continuous variables and chi-square tests for categorical variables

^d^ This category includes 216 black, two Asian Indian/Pakistani, two Asian/Pacific Islander, and two unspecified race in the ET initiation model, with one fewer Asian/Pacific Islander in the ET continuation model

^e^ Other insurance status includes no insurance or self-pay

^f^ Change in menopausal-symptom severity mean scores from patient’s first reported use of ET to 12-18 month follow-up of ET continuation among patients who had used ET within 12 months of definitive surgery

**Table 3:** Variables associated with ET initiation by the 12-month interview and ET continuation for at least 12 months after initiation in the pooled sample

	ET initiation^a^	ET continuation^b^
Independent variables	aOR (95% CI) (*n* = 533)	aOR (95% CI) (*n* = 410)
Race		
White	Ref.	Ref
Black/Other^c^	0.66 (0.38-1.13)	0.85 (0.39-1.85)
Age, years	1.01 (0.98-1.03)	0.97 (0.93-1.00)
Insurance status		
Private	Ref.	Ref
Public only or other^d^	0.94 (0.52-1.71)	1.12 (0.47-2.69)
Chemotherapy		
No	Ref.	Ref
Yes	3.47 (1.77-6.78)^*^	1.23 (0.57-2.66)
Radiation Therapy		
No	Ref.	Ref
Yes	3.94 (2.47-6.31)^*^	1.42 (0.70-2.89)
Diabetes		
No	Ref.	Ref
Yes	0.50 (0.27-0.91)^*^	0.84 (0.34-2.04)
Obesity		
No (BMI < 30 kg/m^2^)	Ref.	Ref
Yes (BMI ≥ 30 kg/m^2^)	1.12 (0.67-1.88)	1.03 (0.52-2.03)
Elevated depressed mood		
No (CESD < 16)	Ref.	Ref
Yes (CESD ≥16)	0.79 (0.41-1.50)	0.46 (0.19-1.11)
Menopausal-symptom severity	1.35 (0.99-1.85)	0.67 (0.45-0.98)^*^
Change in menopausal-symptom severity mean scores^e^	–	1.04 (0.66-1.62)

*ET* endocrine therapy, *BMI* body mass index, *aOR* adjusted odds ratio, *CI* confidence interval, *CESD* Center for Epidemiologic Studies Depression scale

^a^ Using diabetes, obesity, elevated depressed mood, and menopausal symptom data from the interview prior to first report of ET initiation. For non-initiators and patients who first reported ever taking ET at baseline, the baseline measures of these four variables were used

^b^ Using diabetes, obesity, elevated depressed mood, and menopausal symptom data from the interview when ET initiation was first reported

^c^ This category includes 216 black, two Asian Indian/Pakistani, two Asian/Pacific Islander, and two unspecified race in the ET initiation model, with one fewer Asian/Pacific Islander in the ET continuation model

^d^ Other insurance status includes no insurance or self-pay

^e^ Change in menopausal-symptom severity mean scores from patient’s first reported use of ET to 12-18 month follow-up of ET continuation among patients who had used ET within 12 months of definitive surgery

^*^ *P* < 0.05

All participants had non-metastatic breast cancer (24.6% ductal carcinoma in situ, 67.9% early-invasive breast cancer, and 7.5% locally advanced breast cancer). Table 2 presents characteristics of 533 participants in the pooled sample by ET initiation and continuation. Average age was 58.1 years; 41.6% self-identified as black/Asian/unspecified race (40.5% black and 1.1% Asian or unspecified), 42.4% were obese, 16.1% had diabetes, and 20.8% had elevated depressed mood. Additionally, 362 (67.9%) received breast-conserving surgery (BCS), 28.7% received chemotherapy, and 69.0% received radiation therapy. Overall, 81.4% reported initiating ET. A lower proportion of patients with (vs. without) diabetes initiated ET (70.9% vs. 83.4%). Among the 410 patients who initiated ET by the 12-month interview, 90.2% continued ET for at least 12 months.

As shown in Table 3, patients who received chemotherapy (aOR 3.47; 95% CI 1.77-6.78) and radiation therapy (aOR 3.94; 95% CI 2.47-6.31) were each more likely to have initiated ET, but patients with diabetes were less likely to have initiated ET (aOR 0.50; 95% CI 0.27-0.91). Among patients who initiated ET, those who reported more severe menopausal symptoms were less likely to continue ET for at least 12 months (aOR 0.67; 95% CI 0.45-0.98). None of the other variables, including diabetes, depressed mood, obesity, and change in menopausal-symptom severity, was independently associated with ET continuation. However, in sensitivity analyses stratified by race comparing results for only white, only black, and only black/other patients, we observed a significant inverse association between elevated depressed mood and ET continuation among white patients (aOR 0.24; 95% CI 0.07-0.78), but not in either model that included black patients (Table 4).

**Table 4:** Variables associated with endocrine therapy (ET) continuation for at least 12 months after initiation, stratified by race

	aOR (95% CI)^a^		
Independent variables	White (*n* = 248)	Black^b^ (*n* = 157)	Black/Other (*n* = 162)
Age, years	0.96 (0.92-1.01)	0.98 (0.92-1.04)	0.98 (0.92-1.04)
Insurance status			
Private	Ref	Ref	Ref
Public only or other^c^	5.87 (0.36-94.67)	0.54 (0.18-1.57)	0.54 (0.19-1.58)
Chemotherapy			
No	Ref	Ref	Ref
Yes	1.16 (0.39-3.46)	1.58 (0.54-4.58)	1.58 (0.54-4.57)
Radiation Therapy			
No	Ref	Ref	Ref
Yes	1.62 (0.66-3.99)	1.12 (0.35-3.55)	1.16 (0.37-3.68)
Diabetes			
No	Ref	Ref	Ref
Yes	0.49 (0.13-1.87)	1.23 (0.39-3.87)	1.19 (0.38-3.74)
Obesity			
No (BMI < 30 kg/m^2^)	Ref	Ref	Ref
Yes (BMI ≥ 30 kg/m^2^)	1.20 (0.45-3.19)	0.85 (0.32-2.26)	0.83 (0.31-2.19)
Elevated depressed mood			
No (CESD < 16)	Ref	Ref	Ref
Yes (CESD ≥16)	0.24 (0.07-0.78)^*^	0.96 (0.28-3.27)	0.94 (0.28-3.23)
Menopausal-symptom severity	0.66 (0.41-1.07)	0.72 (0.39-1.32)	0.70 (0.38-1.30)
Change in menopausal-symptom severity mean scores^d^	0.77 (0.42-1.43)	1.42 (0.74-2.73)	1.40 (0.73-2.69)

*BMI* body mass index, *aOR* adjusted odds ratio, *CI* confidence interval, *CESD* Center for Epidemiologic Studies Depression scale

^a^ Using diabetes, obesity, elevated depressed mood, and menopausal symptom data from the interview when ET initiation was first reported

^b^ Five patients (two Asian Indian/Pakistani, one Asian/Pacific Islander, and two unspecified because they did not respond to the question about race) were excluded from this sensitivity analysis stratified by race to compare the associations between ET continuation and variables of interest within each racial group

^c^ Other insurance status includes no insurance or self-pay

^d^ Change in menopausal-symptom severity mean scores from patient’s first reported use of ET to 12-18 month follow-up of ET continuation among patients who had used ET within 12 months of definitive surgery

^*^ *P* < 0.05

We conducted another sensitivity analysis to explore if we over or underestimated continuation based on the way patients with missing data were classified as continuing or discontinuing ET. Of the 410 patients who had initiated ET (99 initiated ET at baseline, 234 at 6 months, 77 at 12 months), 15 patients with missing data (six between the baseline and 12-month interviews, six between the 6-month and 24-month interviews, and three between the 12-month and 24-month interviews) were excluded, as we could not confirm that they continued ET for at least 12 months; moreover, eight patients who temporarily stopped ET were reassigned to the discontinued group. There were no significant changes in aORs of ET continuation for the independent variables of interest (Supplementary Table 5).

## Discussion

We examined the associations of diabetes, obesity, depressed mood, and menopausal-symptom severity with ET initiation and continuation in women with HR-positive non-metastatic breast cancer using longitudinal data. We observed that patients with diabetes were significantly less likely to initiate ET within 12 months of enrollment, and patients reporting more severe menopausal symptoms at the time of ET initiation were significantly less likely to have continued use for at least 12 months.

Breast cancer patients with diabetes have poorer outcomes than patients without diabetes [9, 43, 44]. A higher risk of cancer-specific mortality and all-cause mortality in breast cancer patients with diabetes might be due to underutilization of chemotherapy [9, 43, 44] and radiotherapy [9, 44]. Little is known about an independent contribution of comorbid diabetes to ET use. Studies reported that patients with more comorbidities were less likely to initiate ET and continue to use it [2, 7, 12]. However, these studies did not specifically examine the association between diabetes and ET use. A population-based Dutch study of 9725 breast cancer patients examined receipt of various cancer treatments in association with diabetes; patients with diabetes received less aggressive treatment than patients without diabetes [11]. In that study, age was an effect modifier of the association between diabetes and cancer treatment. Patients < 65 years old with (vs. without) diabetes were less likely to have received adjuvant chemotherapy and more likely to have received surgery and ET; however, patients ≥65 years old with (vs. without) diabetes were less likely to have received radiation therapy as older patients with diabetes were also less likely to have received BCS [11]. Due to a small sample size, because we limited our sample to patients with HR-positive tumors, we could not stratify by age in this study. Various diabetes-related complications may explain the underutilization of recommended cancer treatments [10, 11]. Studies demonstrated increased risk of complications from chemotherapy in breast cancer patients with diabetes [43, 45], thus, these patients may be similarly concerned about increased risk of complications from ET and may explain the observed lower ET use among patients with (vs. without) diabetes. An increased risk of venous thrombosis after tamoxifen use [46–48] may also influence diabetic patient’s ET decision making. Given the established benefits of ET for HR-positive breast cancer and the high prevalence of diabetes among breast cancer patients [49, 50], early patient-provider discussions about breast cancer treatment decisions in the context of diabetes management [51] and increased risk for poorer outcomes [52, 53] are recommended to promote ET initiation in patients with diabetes. The demands of self-management for multiple chronic conditions may be challenging to patients with diabetes for whom ET is recommended.

We found that patients who reported more severe menopausal symptoms at the interview prior to ET initiation (or at baseline if ET initiation was reported then) were less likely to continue ET. While 70-80% of HR-positive breast cancer patients are reported to receive ET within 12 months of diagnosis [2, 4, 6], a large proportion of ET users discontinue use due to side effects such as menopausal symptoms [16, 54–57]. Patients often experience severe menopausal symptoms shortly after initiating ET [55, 56], which may negatively affect ET continuation, especially among younger women who may experience menopausal symptoms for the first time [16]. Hot flashes newly treated after ET initiation were found to be associated with earlier ET discontinuation, whereas pre-existing hot flashes were not [15]. Notably, we did not observe a significant association between ET continuation and change in menopausal-symptom severity after ET initiation. To our knowledge, the independent effects of a multi-item menopausal-symptom severity scale and of change in menopausal-symptom severity on ET continuation have not been reported. However, an RCT examined bother by prior treatment side effects at baseline as well as change in bother by individual ET side effects in association with ET continuation [58], with similar results as ours. Menopausal-symptom severity is a commonly reported barrier to ET adherence [54, 58–60]. However, since studies demonstrated that patients with severe menopausal symptoms may experience a greater benefit from ET [61, 62], timely management of severe menopausal symptoms after ET initiation is needed to promote completion of ET. Various non-hormonal interventions have been recommended to relieve discomfort from menopausal symptoms, including maintaining a healthy weight, engaging in regular physical activity, yoga, relaxation techniques, and acupuncture, and avoiding tobacco and alcohol consumption [63–69]. However, there is a lack of published research examining the impact of non-hormonal interventions on both alleviating menopausal-symptom severity and promoting adherence to ET as recommended. Such studies would make substantial contributions to the literature.

Obesity is a common health condition related to breast cancer [18, 19]. Aromatase inhibitors are less effective in lowering the blood estrogen levels in obese patients than non-obese patients [20–22]. Among tamoxifen users, obese patients have a higher risk of developing endometrial cancer than non-obese patients [23, 24]. For these reasons, there might be an inverse association between obesity and ET use. However, we did not observe a significant association between obesity and either ET initiation or continuation. Since obesity is highly correlated with other comorbidities, including diabetes, simultaneous adjustment for diabetes and obesity might have attenuated the association between obesity and ET use. A multi-institutional study also did not observe a significant association between obesity and ET use [70]. Further analysis using a larger sample would refine our understanding of ET initiation and continuation in obese patients with breast cancer.

Depressed mood is commonly reported by breast cancer patients. Pooled data of 43 breast cancer-patient cohorts indicated that 20% of patients self-reported depressed mood, and depression was particularly high during cancer treatment and decreased afterward [71]. Depressed mood can interrupt individuals’ daily routines including general medication use [72]. A prospective study observed that elevated depressed mood at cancer diagnosis was related to higher risk of discontinuing ET within 12 months of initiation [14]. A cross-sectional study also showed a positive association between depressive symptoms and non-persistence of ET use in 12 months [17]. However, we did not observe a significant association between elevated depressed mood and either ET initiation or continuation. This discrepancy in findings might be due, in part, to differences in study design and patient characteristics, as racial minorities accounted for only 6-11% in the other studies and almost 42% in our study. In our sensitivity analysis stratified by race, only white patients with elevated depressed mood had a lower likelihood of ET continuation. Thus, the high proportion of black patients in our sample might explain the discrepancy between our findings and those of other studies. Sufficiently powered studies with diverse racial/ethnic groups and longer follow-up are warranted to determine the impact of depressed mood on ET continuation.

This study has limitations. First, most patients were recruited from a National Cancer Institute-designated comprehensive cancer center and another academic-medical center in the same U.S. city, so the results might not be generalizable to patients treated in community or rural hospitals. Second, ET initiation and continuation were assessed based on a fixed schedule of study interviews, thus the exact timing of initiation or discontinuation could not be determined. We were unable to assess adherence to ET (taking tamoxifen or aromatase inhibitors as prescribed) due to the lack of reliable prescription data in the medical record. Third, although it is not surprising that women who experienced more severe menopausal symptoms might discontinue ET, our findings were based on a larger, more diverse sample of patients than earlier studies that were limited by smaller and less diverse samples [4, 14, 16, 17]. Importantly, we found that greater menopausal-symptom severity at the interview when ET was first reported, not the change in menopausal-symptom severity after ET initiation, was associated with discontinuation of treatment. Significant associations between ET continuation and change in specific ET side effects also were not observed in a large RCT of anastrozole and exemestane [58]. Fourth, ET use was measured over a 2-year follow-up. Thus, the results might not be applicable to the recommended 5-year continuation of ET. Fifth, the Katz’s validated interview of the Charlson Comorbidity Index does not distinguish between Type I and Type II diabetes per se, which might be associated with ET continuation; thus, we could not account for the potential influence of diabetes type in this analysis. Sixth, household income and medication coverage might be associated with ET use and continuation, as costs for ET medication can be a barrier [73]; however, 6% of participants did not report their annual household income. Instead, as previously reported [74], we used insurance status as an indicator of socioeconomic status, since income and insurance status are correlated. Moreover, higher out-of-pocket cost for other medications, such as diabetes medications, could exacerbate financial barriers to ET initiation and long-term adherence. Side effects of other medications were not measured but could have influenced ET initiation or continuation as well. Lastly, cancer care providers might influence participants’ decisions to use ET [75], but information about the role that providers played in ET decisions was not available.

Despite these limitations, this study provided evidence for the potential roles of common comorbidities, including diabetes and severe menopausal symptoms, in association with ET initiation and continuation among women with non-metastatic breast cancer. Given a high prevalence of pre-existing diabetes and its associated breast cancer mortality risk, understanding the role of potentially modifiable barriers, including patient-provider communication [51, 52, 75], is essential for improving ET use by breast cancer patients with diabetes and their prognosis [52, 76]. Timely and effective management of severe menopausal symptoms in breast cancer patients receiving ET is necessary to facilitate their completion of the recommended course of ET.

## Supplementary Information

**Additional file 1.**

## Data Availability

The datasets generated and analyzed for the current study are not publicly available as data for the trial aims are still being analyzed. The pooled data may be made available from the corresponding author on reasonable request.

## Abbreviations

aOR: Adjusted odds ratio
BCS: Breast-conserving surgery
BMI: Body mass index
CESD: Center for Epidemiologic Studies Depression
CI: Confidence interval
ET: Endocrine Therapy
HR: Hormone-receptor
RCT: Randomized controlled trial
