# Supplementary material for: Factors associated with initiation and continuation of endocrine therapy in women with hormone receptor-positive breast cancer
Source: BMC Cancer. 2022 Aug 1;22:837. doi: 10.1186/s12885-022-09946-x (PMC9341086; doi:10.1186/s12885-022-09946-x)
Supplement: Supplementary file 1 — Additional file 1. [file 12885_2022_9946_MOESM1_ESM.docx]

**Supplementary Content For:**

Factors Associated with Initiation and Continuation of Endocrine Therapy in Women with Hormone Receptor-positive Breast Cancer

**This file includes:**

Supplementary Tables 1 through 5

Supplementary Table 1. Differences between intervention and control groups among black women with newly diagnosed, non-metastatic, hormone receptor-positive breast cancers who participated in the randomized controlled trial (n = 157).

| Independent variables | Intervention  n (%) | Control  n (%) | *P*^a^ |
| --- | --- | --- | --- |
| Total | 70 (44.6) | 87 (55.4) |  |
| Age, years^b^ |  |  |  |
| Mean (SD) | 56.2 (9.6) | 56.3 (10.3) | 0.95 |
| Insurance status^b^ |  |  |  |
| Private | 26 (38.8) | 41 (61.2) | 0.21 |
| Public only or other^c^ | 44 (48.9) | 46 (51.1) |  |
| Chemotherapy^d^ |  |  |  |
| No | 42 (44.7) | 52 (55.3) | 0.98 |
| Yes | 28 (44.4) | 35 (55.6) |  |
| Radiation therapy^d^ |  |  |  |
| No | 15 (42.9) | 20 (57.1) | 0.82 |
| Yes | 55 (45.1) | 67 (54.9) |  |
| Diabetes^b^ |  |  |  |
| No | 52 (46.4) | 60 (53.6) | 0.46 |
| Yes | 18 (40.0) | 27 (60.0) |  |
| Obesity^b^ |  |  |  |
| No (BMI < 30 kg/m^2^) | 27 (43.6) | 35 (56.4) | 0.83 |
| Yes (BMI ≥ 30 kg/m^2^) | 43 (45.3) | 52 (54.7) |  |
| Elevated depressed mood^b^ |  |  |  |
| No (CESD < 16) | 43 (41.8) | 60 (58.2) | 0.32 |
| Yes (CESD ≥ 16) | 27 (50.0) | 27 (50.0) |  |
| Menopausal-symptom severity^b^ |  |  |  |
| Mean (SD) | 1.9 (0.8) | 1.9 (1.0) | 0.67 |
| ET initiation within 12 months |  |  |  |
| No | 16 (55.6) | 20 (44.4) | 0.98 |
| Yes | 54 (55.4) | 67 (44.6) |  |
| ET continuation for at least 12 months |  |  |  |
| No | 11 (52.2) | 12 (47.8) | 0.72 |
| Yes | 42 (56.2) | 54 (43.8) |  |

*SD* standard deviation, *BMI* body mass index, *CESD* Center for Epidemiologic Studies Depression scale, *ET* endocrine therapy.

^a^ Differences by arm were tested using independent *t-*tests for continuous variables and chi-square tests for categorical variables.

^b^ Based on baseline measures.

^c^ Other insurance status includes no insurance or self-payment

^d^ Based on treatment at any time over the study.

Supplementary Table 2. Effects of the intervention on ET initiation and continuation among black women with newly diagnosed, non-metastatic, hormone receptor-positive breast cancers who participated in the randomized controlled trial.

|  | ET initiation^a^ | | | | ET continuation^b^ | | | |
| --- | --- | --- | --- | --- | --- | --- | --- | --- |
| Independent variables | Total  N | Yes  n (%) | No  n (%) | aOR (95% CI) | Total  n | Yes  n (%) | No  n (%) | aOR (95% CI) |
| Total | 157 | 121 (77.1) | 36 (22.9) |  | 111 | 96 (86.5) | 15 (13.5) |  |
| Arm |  |  |  |  |  |  |  |  |
| Intervention | 70 | 54 (77.1) | 16 (22.9) | 0.99 (0.43-2.30) | 48 | 42 (87.5) | 6 (12.5) | 1.17 (0.39-3.46) |
| Control | 87 | 67 (77.0) | 20 (23.0) | Ref. | 63 | 54 (85.7) | 9 (14.3) | Ref. |
| Age, years |  |  |  |  |  |  |  |  |
| Mean (SD) | 56.2 (10.0) | 55.3 (9.8) | 59.4 (10.1) | 1.01 (0.97-1.06) | 55.4 (9.8) | 55.1 (10.0) | 57.6 (8.1) | 0.97 (0.90-1.03) |
| Insurance status |  |  |  |  |  |  |  |  |
| Private | 67 | 51 (76.1) | 16 (23.9) | Ref. | 48 | 44 (91.7) | 4 (8.3) | Ref. |
| Public only or other^c^ | 90 | 70 (77.8) | 20 (22.2) | 0.91 (0.36-2.30) | 63 | 52 (82.5) | 11 (17.5) | 0.47 (0.13-1.67) |
| Chemotherapy |  |  |  |  |  |  |  |  |
| No | 94 | 64 (68.1) | 30 (31.9) | Ref. | 57 | 48 (84.2) | 9 (15.8) | Ref. |
| Yes | 63 | 57 (90.5) | 6 (9.5) | 3.24 (1.16-9.01)^*^ | 54 | 48 (88.9) | 6 (11.1) | 1.31 (0.42-4.07) |
| Radiation therapy |  |  |  |  |  |  |  |  |
| No | 35 | 17 (48.6) | 18 (51.4) | Ref. | 15 | 12 (80.0) | 3 (20.0) | Ref. |
| Yes | 122 | 104 (85.2) | 18 (14.8) | 4.85 (1.93-12.18)^*^ | 96 | 84 (87.5) | 12 (12.5) | 1.66 (0.40-6.83) |
| Diabetes |  |  |  |  |  |  |  |  |
| No | 111 | 91 (82.0) | 20 (18.0) | Ref. | 80 | 69 (86.2) | 11 (13.8) | Ref. |
| Yes | 46 | 30 (65.2) | 16 (34.8) | 0.47 (0.19-1.15) | 31 | 27 (87.1) | 4 (12.9) | 1.26 (0.38-4.19) |
| Obesity |  |  |  |  |  |  |  |  |
| No (BMI < 30 kg/m^2^) | 60 | 48 (80.0) | 12 (20.0) | Ref. | 46 | 40 (87.0) | 6 (13.0) | Ref. |
| Yes (BMI ≥ 30 kg/m^2^) | 97 | 73 (75.3) | 24 (24.7) | 0.65 (0.27-1.57) | 65 | 56 (86.1) | 9 (13.9) | 0.79 (0.26-2.45) |
| Elevated depressed mood |  |  |  |  |  |  |  |  |
| No (CESD < 16) | 101 | 74 (73.3) | 27 (26.7) | Ref. | 75 | 67 (89.3) | 8 (10.7) | Ref. |
| Yes (CESD ≥ 16) | 56 | 47 (83.9) | 9 (16.1) | 1.05 (0.38-2.87) | 36 | 29 (80.6) | 7 (19.4) | 0.80 (0.20-3.21) |
| Menopausal-symptom severity, mean (SD) | 1.9 (0.9) | 2.0 (1.0) | 1.5 (0.6) | 2.10 (1.16-3.81)^*^ | 2.3 (1.0) | 2.3 (1.0) | 2.6 (1.0) | 0.83 (0.42-1.64) |
| Change in menopausal-symptom severity mean score^d^ | - | - | - | **-** | -0.09 (0.82) | -0.04 (0.76) | -0.35 (1.13) | 1.24 (0.64-2.40) |

*ET* endocrine therapy, *SD* standard deviation, *BMI* body mass index, *aOR* adjusted odds ratio, *CI* confidence interval, *CESD* Center for Epidemiologic Studies Depression scale.

^a^ ET initiated by the 12-month interview. Adjusted logistic regression models included all variables listed. Diabetes, obesity, elevated depressed mood, and menopausal symptom data were derived from the interview prior to first report of ET initiation. For patients who did not report ET use by the 12-month interview and patients who first reported ever taking ET at baseline, the baseline measures of these four variables were used.

^b^ ET continued for at least 12 months after initiation. Adjusted logistic regression models included all variables listed. Diabetes, obesity, elevated depressed mood, and menopausal symptom data were derived from the interview when ET initiation was first reported. Of 121 patients who initiated ET within first 12 months of the study, two were excluded due to missing data for obesity (n = 1) and diabetes (n = 1), and eight were excluded due to missing data for menopausal symptom severity at follow-up interviews.

^c^ Other insurance status includes no insurance or self-payment.

^d^ Change in menopausal-symptom severity mean scores from patient’s first reported use of ET to longest follow-up of ET continuation among patients who had used ET at least 12 months.

^*^ *P* < 0.05.

Supplementary Table 3. The associations of exposure variables of interest with the likelihoods of ET initiation and continuation in women with newly diagnosed, non-metastatic, hormone receptor-positive breast cancers who participated in the cohort study

|  | ET initiation^a^ | | | | ET continuation^b^ | | | |
| --- | --- | --- | --- | --- | --- | --- | --- | --- |
| Independent variables | Total  N | Yes  n (%) | No  n (%) | aOR (95% CI) | Total  n | Yes  n (%) | No  n (%) | aOR (95% CI) |
| Total | 376 | 313 (83.2) | 63 (16.8) |  | 299 | 274 (91.6) | 25 (8.4) |  |
| Race |  |  |  |  |  |  |  |  |
| White | 311 | 259 (83.3) | 52 (16.7) | Ref. | 248 | 226 (91.1) | 22 (8.9) | Ref. |
| Black/Other | 65 | 54 (83.1) | 11 (16.9) | 1.02 (0.46-2.26) | 51 | 48 (94.1) | 3 (5.9) | 1.03 (0.32-3.33) |
| Age, years |  |  |  |  |  |  |  |  |
| Mean (SD) | 58.9 (10.7) | 58.7 (10.3) | 59.5 (12.8) | 1.01 (0.98-1.04) | 58.7 (10.2) | 58.6 (10.2) | 60.3 (10.0) | 0.97 (0.93-1.02) |
| Insurance status |  |  |  |  |  |  |  |  |
| Private | 322 | 269 (83.5) | 53 (16.5) | Ref. | 259 | 235 (90.7) | 24 (9.3) | Ref. |
| Public only or other^c^ | 54 | 44 (81.5) | 10 (18.5) | 0.90 (0.39-2.06) | 40 | 39 (97.5) | 1 (2.5) | 3.40 (0.63-18.43) |
| Chemotherapy |  |  |  |  |  |  |  |  |
| No | 286 | 229 (80.1) | 57 (19.9) | Ref. | 218 | 198 (90.8) | 20 (9.2) | Ref. |
| Yes | 90 | 84 (93.3) | 6 (6.7) | 3.66 (1.50-8.93)^*^ | 81 | 76 (93.8) | 5 (6.2) | 1.49 (0.53-4.15) |
| Radiation therapy |  |  |  |  |  |  |  |  |
| No | 130 | 91 (70.0) | 39 (30.0) | Ref. | 86 | 77 (89.5) | 9 (10.5) | Ref. |
| Yes | 246 | 222 (90.2) | 24 (9.8) | 3.74 (2.11-6.61)^*^ | 213 | 197 (92.5) | 16 (7.5) | 1.57 (0.68-3.63) |
| Diabetes |  |  |  |  |  |  |  |  |
| No | 336 | 282 (83.9) | 54 (16.1) | Ref. | 270 | 248 (91.8) | 22 (8.2) | Ref. |
| Yes | 40 | 31 (77.5) | 9 (22.5) | 0.61 (0.26-1.48) | 29 | 26 (89.7) | 3 (10.3) | 0.73 (0.21-2.56) |
| Obesity |  |  |  |  |  |  |  |  |
| No (BMI < 30 kg/m^2^) | 247 | 201 (81.4) | 46 (18.6) | Ref. | 192 | 175 (91.1) | 17 (8.9) | Ref. |
| Yes (BMI ≥ 30 kg/m^2^) | 129 | 112 (86.8) | 17 (13.2) | 1.45 (0.74-2.84) | 107 | 99 (92.5) | 8 (7.5) | 1.00 (0.42-2.38) |
| Elevated depressed mood |  |  |  |  |  |  |  |  |
| No (CESD < 16) | 321 | 269 (83.8) | 52 (16.2) | Ref. | 264 | 244 (92.4) | 20 (7.6) | Ref. |
| Yes (CESD ≥ 16) | 55 | 44 (80.0) | 11 (20.0) | 0.66 (0.29-1.52) | 35 | 30 (85.7) | 5 (14.3) | 0.40 (0.12-1.11) |
| Menopausal-symptom severity, mean (SD) | 1.7 (0.8) | 1.7 (0.8) | 1.7 (0.9) | 1.08 (0.75-1.55) | 2.0 (0.9) | 1.9 (0.9) | 2.3 (1.1) | 0.63 (0.40-1.00) |
| Change in menopausal-symptom severity mean score^d^ | - | - | - | - | 0.10 (0.71) | 0.10 (0.71) | 0.09 (0.79) | 0.85 (0.47-1.51) |

*ET* endocrine therapy, *SD* standard deviation, *BMI* body mass index, *aOR* adjusted odds ratio, *CI* confidence interval, *CESD* Center for Epidemiologic Studies Depression scale.

^a^ ET initiated by the 12-month interview. Adjusted logistic regression models included all variables listed. Diabetes, obesity, elevated depressed mood, and menopausal symptom data were derived from the interview prior to first report of ET initiation. For patients who did not report ET use by the 12-month interview and patients who first reported ever taking ET at baseline, the baseline measures of these four variables were used.

^b^ ET continued for at least 12 months after initiation. Adjusted logistic regression models included all variables listed. Diabetes, obesity, elevated depressed mood, and menopausal symptom data were derived from the interview when ET initiation was first reported. Of 313 patients who initiated ET within first 12 months of the study, three were excluded due to missing data for obesity and 11 were excluded due to missing data for menopausal symptom severity at follow-up interviews.

^c^ Other insurance status includes no insurance or self-payment.

^d^ Change in menopausal-symptom severity mean scores from patient’s first reported use of ET to longest follow-up of ET continuation among patients who had used ET at least 12 months.

^*^ *P* < 0.05.

Supplementary Table 4. Variables associated with ET initiation by the 12-month interview and ET continuation for at least 12 months after initiation in white and only black women with hormone receptor-positive breast cancer in the pooled sample.

|  | ET initiation^a^ | ET continuation^b^ |
| --- | --- | --- |
| Independent variables | aOR (95% CI)  (n = 527) | aOR (95% CI)  (n = 405) |
| Race |  |  |
| White | Ref. | Ref |
| Black | 0.66 (0.38-1.15) | 0.83 (0.38-1.80) |
| Age, years | 1.01 (0.99-1.03) | 0.97 (0.93-1.00) |
| Insurance status |  |  |
| Private | Ref. | Ref |
| Public only or other^c^ | 0.94 (0.52-1.70) | 1.12 (0.46-2.70) |
| Chemotherapy |  |  |
| No | Ref. | Ref |
| Yes | 3.44 (1.76-6.72)^*^ | 1.23 (0.57-2.66) |
| Radiation therapy |  |  |
| No | Ref. | Ref |
| Yes | 3.83 (2.39-6.12)^*^ | 1.41 (0.69-2.86) |
| Diabetes |  |  |
| No | Ref. | Ref |
| Yes | 0.50 (0.76-0.92)^*^ | 0.85 (0.35-2.04) |
| Obesity |  |  |
| No (BMI < 30 kg/m^2^) | Ref. | Ref |
| Yes (BMI ≥ 30 kg/m^2^) | 1.10 (0.66-1.84) | 1.03 (0.52-2.04) |
| Elevated depressed mood |  |  |
| No (CESD < 16) | Ref. | Ref |
| Yes (CESD ≥ 16) | 0.80 (0.42-1.52) | 0.47 (0.19-1.13) |
| Menopausal-symptom severity, mean score | 1.35 (0.98-1.84) | 0.67 (0.46-0.99) |
| Change in menopausal-symptom severity mean score^d^ | - | 1.04 (0.66-1.63) |

*ET* endocrine therapy, *BMI* body mass index, *aOR* adjusted odds ratio, *CI* confidence interval, *CESD* Center for Epidemiologic Studies Depression scale.

^a^ Using diabetes, obesity, elevated depressed mood, and menopausal symptom data from the interview prior to first report of ET initiation. For non-initiators and patients who first reported ever taking ET at baseline, the baseline measures of these four factors were used.

^b^ Using diabetes, obesity, elevated depressed mood, and menopausal symptom data from the interview when ET initiation was first reported.

^c^ Other insurance status includes no insurance or self-payment.

^d^ Change in menopausal-symptom severity mean scores from patient’s first reported use of ET to longest follow-up of ET continuation among patients who had used ET at least 12 months.

^*^ *P* < 0.05.

Supplementary Table 5. Sensitivity analysis to identify variables associated with ET continuation for at least 12 months after initiation, reassigning 8 patients who temporarily stopped using ET after initiation to the discontinued group and excluding the 15 patients who were missing current-use data during the relevant 12-month interval.

|  |  |
| --- | --- |
| Independent variables | aOR (95% CI)^a^  (n = 395) |
| Race |  |
| White | Ref |
| Black/Other^b^ | 0.96 (0.45-2.03) |
| Age, years | 0.98 (0.95-1.02) |
| Insurance status |  |
| Private | Ref |
| Public only or other^c^ | 1.49 (0.59-3.76) |
| Chemotherapy |  |
| No | Ref |
| Yes | 1.77 (0.79-3.96) |
| Radiation therapy |  |
| No | Ref |
| Yes | 1.36 (0.67-2.78) |
| Diabetes |  |
| No | Ref |
| Yes | 0.82 (0.34-2.01) |
| Obesity |  |
| No (BMI < 30 kg/m^2^) | Ref |
| Yes (BMI ≥ 30 kg/m^2^) | 0.94 (0.48-1.77) |
| Elevated depressed mood |  |
| No (CESD < 16) | Ref |
| Yes (CESD ≥ 16) | 0.70 (0.28-1.77) |
| Menopausal-symptom severity, mean score | 0.65 (0.45-0.96)^*^ |
| Change in menopausal-symptom severity mean score^d^ | 1.42 (0.90-2.24) |

*ET* endocrine therapy, *BMI* body mass index, *aOR* adjusted odds ratio, *CI* confidence interval, *CESD* Center for Epidemiologic Studies Depression scale.

^a^ Using diabetes, obesity, elevated depressed mood, and menopausal symptom data from the interview when ET initiation was first reported.

^b^ This group included 148 black, two Asian Indian/Pakistani, one Asian/Pacific Islander, and two patients who did not respond to this question.

^c^ Other insurance status includes no insurance or self-payment.

^d^ Change in menopausal-symptom severity mean scores from patient’s first reported use of ET to longest follow-up of ET continuation among patients who had used ET at least 12 months.

^*^ *P* < 0.05.
